# Omalizumab-based therapy in normocomplementemic urticarial vasculitis: a retrospective case series of 18 patients^؆^

**DOI:** 10.1016/j.abd.2026.501424

**Published:** 2026-08-06

**Authors:** Melek Aslan Kayiran, Kubra Akdemir Yucel, Bengu Cobanoglu Simsek, Mehmet Salih Gurel

**Affiliations:** a Department of Dermatology, Faculty of Medicine, Goztepe Training and Research Hospital, Istanbul Medeniyet University, Istanbul, Turkey; b Department of Pathology, Faculty of Medicine, Goztepe Training and Research Hospital, Istanbul Medeniyet University, Istanbul, Turkey

**Keywords:** IgE, Omalizumab, Treatment outcome, Urticaria, Cutaneous leukocytoclastic vasculitis

## Abstract

**Background:**

Urticarial Vasculitis (UV) is a rare immune-complex–mediated vasculitis characterized by urticarial lesions lasting ≥24 hours and small-vessel inflammation. Management is challenging, and evidence for omalizumab, an anti-IgE monoclonal antibody, remains limited.

**Objectives:**

To evaluate clinical and laboratory outcomes and safety of omalizumab-based therapy in patients with clinicopathologically diagnosed normocomplementemic UV.

**Methods:**

This retrospective study included 18 patients with clinicopathologically diagnosed normocomplementemic UV treated with omalizumab. Demographics, disease duration, comorbidities, and laboratory parameters (C-Reactive Protein [CRP], Erythrocyte Sedimentation Rate [ESR], total Immunoglobulin E [IgE]) and Urticaria Activity Score over 7 days (UAS7) were assessed before and after treatment.

**Results:**

Eighteen patients (14 females, 4 males) with a mean age of 47.3 ± 16.8 years were included. Mean disease duration was 15.8 ± 10.8 months, and mean omalizumab treatment duration was 14.8 ± 12.0 months. Mean UAS7 decreased significantly from 25.3 ± 7.7 to 1.2 ± 3.4 (p < 0.001). CRP also decreased significantly (10.7 ± 17.2 to 3.6 ± 3.3 mg/L; p = 0.002), while the reduction in ESR was not significant (18.2 ± 26.5 to 10.9 ± 7.8 mm/h; p = 0.124). Total IgE increased after treatment (451.6 ± 1075.5 to 496.4 ± 621.2 IU/mL; p = 0.010). Complete remission occurred in 17 patients (94%). No treatment-related adverse events were observed.

**Study limitations and conclusions:**

Limitations include retrospective design, small sample size, lack of a control group, and concomitant therapies. Omalizumab was associated with marked clinical improvement and favorable safety in normocomplementemic UV, supporting a potential role for IgE-mediated mechanisms.

## Introduction

Urticarial vasculitis (UV) is an immune complex-mediated vasculitis characterized by erythematous, raised plaques and papules affecting the small blood vessels of the skin. The primary distinguishing features of UV compared with classical spontaneous urticaria include lesions that typically persist for at least 24 hours and often resolve with residual hyperpigmentation. Although UV lesions may be pruritic, they are more frequently painful, with a stinging or burning sensation. UV affects women more frequently than men, and although it can occur at any age, it is most observed in the fourth decade of life.^1^, ^2^ The prevalence of urticarial vasculitis among patients presenting with chronic urticaria has been estimated to range from 3% to 20%, although reliable prevalence data remain limited.^3^ Common laboratory findings in urticarial vasculitis include elevated erythrocyte sedimentation rate (ESR). Although reduced serum complement levels may also be observed, most patients are normocomplementemic, and approximately 20% of cases may be hypocomplementemic.^3^, ^4^ In most cases, the etiological factors remain unidentified. However, infection is the most frequently identified cause, with other potential contributors including drugs, neoplastic diseases, and autoimmune conditions.^4^, ^5^

UV is classified as either normocomplementemic urticarial vasculitis (NUV) or hypocomplementemic urticarial vasculitis (HUV). The prognosis of NUV is generally more favorable, with fewer systemic complications. In contrast, HUV is associated with a higher risk of systemic involvement and complications.^4^, ^6^ Various treatment approaches have been explored for UV, including oral antihistamines (OAH) for mild cases; anti-inflammatory agents such as colchicine, dapsone, and hydroxychloroquine; immunosuppressants such as corticosteroids, azathioprine, and cyclosporine; and biological therapies, including omalizumab, for moderate to severe cases or those with systemic involvement.^7^, ^8^, ^9^

Omalizumab is a humanized anti-Immunoglobulin (Ig) E monoclonal antibody used in the management of chronic spontaneous urticaria (CSU). Studies have shown that omalizumab significantly reduces the severity and manifestations of CSU, decreasing the need for additional pharmacological agents and improving quality of life.^10^, ^11^ The limited evidence on the efficacy of omalizumab in the treatment of UV increases the importance of this retrospective study. This study aims to evaluate the effectiveness and outcomes of omalizumab treatment in patients with UV.

## Materıals and methods

After obtaining approval from the local ethics committee, this retrospective study was conducted at the outpatient dermatology clinic of a tertiary care center. Ethics committee approval was obtained (approval nº 01/08/2024/E-10840098-202.3.02-4635). Due to the retrospective design of the study and the use of anonymized data, the requirement for written informed consent was waived by the ethics committee. Medical records of patients with clinicopathologically diagnosed UV who received omalizumab treatment between January 1, 2020, and August 1, 2024, were reviewed. The biopsies were performed in patients presenting with urticarial plaques lasting longer than 24 hours and resolving with residual purpura or hyperpigmentation, accompanied by burning and stinging sensations. The diagnosis of UV was established based on clinicopathological correlation in patients with histopathological findings compatible with urticarial vasculitis. Patients with incomplete records, hypocomplementemia, or alternative vasculitides were excluded.

Socio-demographic characteristics, disease duration, previously administered treatments, duration and dosage of omalizumab, treatment response, pre- and post-treatment laboratory data, and Urticarial Activity Scores over the last 7 days (UAS7) at the time of follow-up were retrieved from patient records and documented. Data were analyzed using SPSS (SPSS for Windows, version 21; IBM Corp, Armonk, NY, USA). Continuous variables are presented as mean ± standard deviation. Normality was assessed using the Shapiro-Wilk test. Paired *t*-tests were used for normally distributed parameters. A p-value < 0.05 was considered statistically significant.

## Results

A total of 18 patients diagnosed with UV who received omalizumab treatment in the specified period were included. The mean age at diagnosis was 47.3 ± 16.8 years (range, 25–77 years). The mean disease duration was 15.8 ± 10.8 months (range, 4–48 months). Six patients (33%) had a history of at least one allergic disease. Among them, asthma was detected in four patients (22%), allergic rhinitis in one patient (6%), and atopic dermatitis in one patient (6%). Eight patients (44%) had previously been diagnosed with CSU for the same rash before being referred to our clinic. Four of them had been treated with OAH without any response. The other four showed only a partial response to both OAH and omalizumab.

Two patients had autoimmune diseases, including type 1 diabetes mellitus and autoimmune thyroiditis. Six patients had at least one systemic comorbidity, such as hypertension, hypercholesterolemia, or Behçet’s disease.

Histopathological examination revealed epidermal thinning. There was perivascular lymphocytic, neutrophilic, and eosinophilic infiltration in the dermis, along with thickened vessel walls and focal erythrocyte extravasation. Direct immunofluorescence demonstrated fibrinogen deposition in the vessel walls of eleven patients. Although some specimens showed features compatible with urticarial vasculitis, classic findings such as leukocytoclasia and fibrinoid necrosis of the vessel wall were not consistently demonstrated in all cases. Therefore, the diagnosis was established based on clinicopathological correlation.

Table 1 summarizes the sociodemographic characteristics and baseline and post-treatment laboratory and clinical parameters, including C-Reactive protein (CRP), Erythrocyte Sedimentation Rate (ESR), total Immunoglobulin E (IgE), and UAS7. At treatment initiation, elevated CRP was observed in seven patients (39%) (mean CRP among these patients: 13.7 ± 4.4 mg/L) and elevated ESR in four patients (22%) (mean ESR among these patients: 49.5 ± 40.8 mm/h); overall baseline CRP and ESR values for the entire cohort are presented in Table 1. Total IgE levels were above the normal reference range (< 100 IU/mL) in ten patients (56%).

**Table 1:** Demographic, clinical, and laboratory characteristics of the patients.

Demographic, Clinical, and Laboratory Features	Values
Female / Male	14 (78%) / 4 (22%)
Age (mean ± SD)	47.3 ± 16.8 years (range: 25–77)
Smokers / Non-smokers	3 (17%) / 15 (83%)
History of atopy	6 / 18 (33%)
History of autoimmune disease	2 / 18 (11%)
History of systemic disease	6/18 (33%)
Disease duration (mean ± SD)	15.8 ± 10.8 months (range: 4–48)
Duration of omalizumab treatment (mean ± SD)	14.8 ± 12.0 months (range: 2–48)
Total number of omalizumab doses (mean ± SD)	11.2 ± 10.9 (range: 2–48)
UAS7 before / After treatment (mean ± SD)	25.3 ± 7.7 / 1.2 ± 3.4 (p < 0.001)
CRP (mg/L) before / After treatment (mean ± SD)	10.7 ± 17.2 / 3.6 ± 3.3 mg/L (p = 0.002)
ESR (mm/h) before / After treatment (mean ± SD)	18.2 ± 26.5 / 10.9 ± 7.8 (p = 0.124)
Total IgE (IU/mL) before / After treatment (mean ± SD)	451.6 ± 1075.5 / 496.4 ± 621.2 (p = 0.010)

UAS7, Urticaria Activity Score-7; CRP, C-Reactive Protein; ESR, Erythrocyte Sedimentation Rate; IgE, Immunoglobulin-E; SD, Standard Deviation.

One patient had hypercholesterolemia/hypertriglyceridemia, one had elevated liver enzymes, and one had iron deficiency anemia. Normocomplementemia was observed in all eighteen patients. Additionally, Antinuclear Antibody (ANA) positivity was detected in one case during diagnostic evaluation. Four patients reported a history of infection that required treatment with penicillin or quinolone group antibiotics as a triggering factor prior to lesion onset. Clinical symptoms included arthralgia in six patients (33%), fever in 4 (22%), abdominal pain in 3 (17%), fatigue in 2 (11%), and nausea and vomiting in one patient (6%).

Before omalizumab initiation, all patients had been treated with OAHs (including rupatadine, cetirizine, desloratadine, and bilastine), used at standard or up-dosed regimens up to fourfold, similar to chronic urticaria treatment. Seven received systemic corticosteroids, and three had colchicine. Four patients also used omalizumab for a short period prior to inclusion in this study. All patients who did not achieve satisfactory outcomes from prior treatments were started on subcutaneous omalizumab, 300 mg every four weeks. Of 18 patients who received omalizumab with OAH, eight achieved complete remission and stayed symptom-free during follow-up. In seven, the addition of colchicine (0.5 mg twice daily) to omalizumab led to full lesion resolution. One of these required an increase in omalizumab to 450 mg after an inadequate response to 300 mg; this resulted in marked improvement within a week. Two patients achieved remission with omalizumab alone. One patient achieved only partial clinical improvement despite the addition of adjunctive therapies, including doxepin, colchicine, and cyclosporine. Representative clinical photographs before and after omalizumab treatment are shown in Fig. 1.

**Fig. 1:**
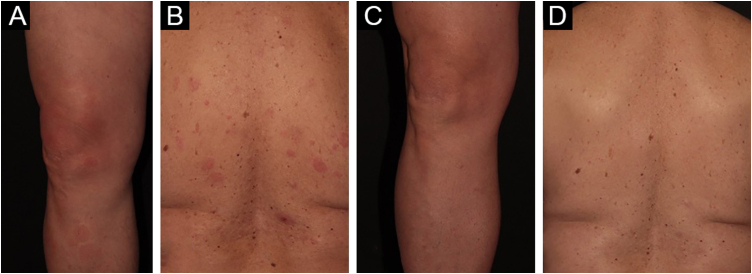
(A–B) Presents erythematous and edematous papules and plaques on the legs and back of a 64-year-old male patient prior to omalizumab treatment. (C–D) Illustrates complete resolution of the lesions following the fifth omalizumab dose during follow-up.

## Dıscussıon

In the present study, UV was more prevalent in female patients, accounting for 78%. The mean age was 47.3 ± 16.8 years (range, 25‒77 years), which is consistent with previous reports indicating a female predominance and a peak incidence in the fourth decade of life.^5^, ^12^ Similarly, another study reported a mean age at disease onset of 42 years. However, while the mean disease duration in that study was four years,^13^ patients in our cohort had a considerably shorter mean disease duration of 15.8 ± 10.8 months.

Atopic comorbidities have been variably reported in patients with UV. In one study, a history of atopy was noted in 8 of 72 patients (11%), although the specific atopic conditions were not detailed.^14^ In contrast, atopic diseases were identified in 6 of 18 patients (33%) in our study, suggesting a relatively higher prevalence. The reported prevalence of UV among patients with Chronic Spontaneous Urticaria (CSU) ranges from 3% to 20% in the literature.^3^ Notably, 8 of 18 patients (44%) in our cohort had a prior diagnosis of CSU, representing a substantially higher rate than previously reported.

Immunopathological findings in UV commonly include immune complex deposition in the vessel walls and at the dermoepidermal junction. Previous studies have demonstrated deposits of IgM, IgG, IgA, complement components C1q, C3, and C4, as well as fibrinogen, in affected skin specimens.^15^ In the present study, direct immunofluorescence revealed fibrinogen deposition in the vessel walls in 11 of 18 patients (61%), supporting the role of immune complex-mediated vascular injury in UV (Fig. 2A–C).

**Fig. 2:**
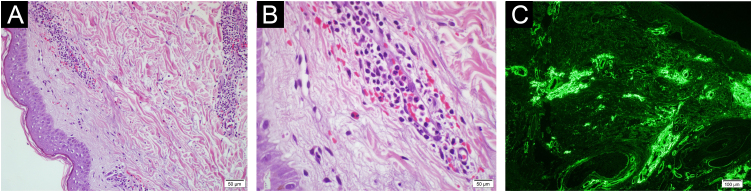
(A–B) Histopathological findings compatible with urticarial vasculitis, showing inflammatory cell infiltration in and around the dermal vessel walls, including neutrophils and eosinophils, together with erythrocyte extravasation (A, Hematoxylin & eosin stain, ×200; B, Hematoxylin & eosin stain, ×600). (C) Direct immunofluorescence showing fibrinogen deposition in the vessel walls. These findings were interpreted in conjunction with the clinical presentation.

Regarding systemic involvement, arthralgia was the most frequently observed extracutaneous symptom in our cohort, consistent with prior studies identifying arthralgia as the most common systemic manifestation of UV.^13^, ^14^ Laboratory abnormalities previously associated with UV include elevated ESR and hypocomplementemia, with increased ESR reported in approximately 75% of cases.^3^ Another study documented low complement levels in 13 of 68 patients (19%).^4^ In contrast, the most frequent laboratory abnormalities observed in our study were elevated total IgE, CRP, and ESR levels. Total IgE levels exceeded the normal reference range (< 100 IU/mL) in 10 patients (56%), elevated CRP levels were detected in 7 patients (39%), and increased ESR values were observed in 4 patients (22%).

Mean ESR decreased from 18.2 ± 26.5 mm/h to 10.9 ± 7.8 mm/h after treatment; however, this change did not reach statistical significance. In contrast, CRP levels showed a significant reduction following treatment (10.7 ± 17.2 vs. 3.6 ± 3.3 mg/L, p = 0.002). The observed decline in CRP may reflect the suppression of IgE-mediated mast cell and basophil activation, leading to attenuation of systemic inflammatory responses. This immunomodulatory effect may, in turn, result in decreased production of proinflammatory cytokines, including Interleukin-6 (IL-6) and Tumour Necrosis Factor-α (TNF-α), which are key drivers of hepatic CRP synthesis. By comparison, ESR is a slower-responding and less specific inflammatory marker influenced by multiple non-inflammatory factors, which may account for the absence of a statistically significant change, particularly in the context of the relatively small sample size.

During the diagnostic evaluation, ANA positivity was detected in one patient. Previous studies have shown that complement levels remain consistently normal in patients with NUV, whereas ANA positivity is observed in approximately 27% of cases, usually at low titers.^4^ Accordingly, the isolated ANA positivity observed in our patient does not contradict the diagnosis of NUV and is most likely a nonspecific serologic finding.

In a previous study, elevated serum total IgE levels ranging from 132 to 542 IU/mL were reported in 4 of 5 patients (80%), exceeding the normal reference range (< 100 IU/mL).^16^ In the present study, total IgE levels ranged from 11 to 4298 IU/mL, with elevated values observed in 10 of 18 patients (56%). Following omalizumab treatment, total IgE levels showed a statistically significant increase (451.6 ± 1075.5 vs. 496.4 ± 621.2 IU/mL, p = 0.010). This increase is unlikely to represent de novo IgE synthesis; rather, it is more plausibly attributable to the prolonged circulation of omalizumab–IgE immune complexes. Consistent with this interpretation, previous studies have demonstrated that omalizumab therapy leads to an increase in measured total IgE levels while effectively reducing free IgE concentrations.^17^

Although the etiology of urticarial vasculitis is often idiopathic, several triggering factors have been implicated, including medications, infections, autoimmune diseases, and malignancies.^3^, ^5^, ^9^ In the present study, potential triggers identified during clinical evaluation included antecedent infections and prior exposure to antibiotics, particularly penicillins and quinolones. Four patients reported a history of infection accompanied by antibiotic use before the onset of cutaneous lesions. Additionally, one patient was diagnosed with pulmonary tuberculosis during the etiological work-up.

In a recent prospective study, urticarial vasculitis was shown to differ from chronic spontaneous urticaria in time to diagnosis, clinical presentation, and need for anti-inflammatory treatment, underscoring the distinct therapeutic challenges of this condition.^18^ Omalizumab, a humanized anti-IgE monoclonal antibody, has been shown to effectively reduce symptom severity and frequency in chronic spontaneous urticaria, thereby decreasing the need for additional medications and improving patients’ quality of life.^10^, ^11^ Although the precise role of IgE in the pathogenesis of urticarial vasculitis remains incompletely understood, accumulating evidence from case reports and small clinical series supports the efficacy of omalizumab in disease control.^16^, ^19^, ^20^ In previous studies, the most commonly reported adverse effects of omalizumab included headache, fatigue, and injection-site reactions.^21^ Notably, no treatment-related adverse events were observed in our cohort.

In the present study, mean UAS7 scores decreased markedly from 25.3 ± 7.7 before treatment to 1.2 ± 3.4 following omalizumab therapy (p < 0.001), indicating substantial clinical improvement. This pronounced reduction in disease activity highlights the potential of omalizumab as an effective therapeutic option for UV in real-world clinical practice. While the retrospective design, limited sample size, concomitant treatments, and lack of a control group should be acknowledged, these findings underscore the need for prospective, controlled studies to further define the role of omalizumab in the management of urticarial vasculitis. In addition to its potential efficacy, the favorable safety profile of omalizumab may represent an important advantage in the management of normocomplementemic urticarial vasculitis, particularly when compared with long-term corticosteroid or immunosuppressive therapies.

Limitations of this study include its retrospective design and the relatively small sample size, which may limit generalizability and preclude robust subgroup analyses. In addition, the absence of a control group prevents definitive conclusions regarding causality and treatment effect. Concomitant medications (e.g., antihistamines and add-on anti-inflammatory/immunosuppressive agents in some patients) may have confounded the observed clinical and laboratory improvements. Finally, post-treatment assessments were performed at follow-up visits in routine clinical practice, and the timing of “post-treatment” measurements may have varied across patients, potentially introducing heterogeneity in outcome evaluation.

## Conclusion

Omalizumab appears to be a safe and effective therapeutic option for patients with urticarial vasculitis, leading to marked clinical improvement and a significant reduction in disease activity. The substantial decrease in UAS7 scores observed in this cohort supports its clinical efficacy, while the absence of treatment-related adverse events highlights its favorable safety profile. In addition, the observed changes in inflammatory parameters suggest a potential immunomodulatory effect of omalizumab. Although the precise role of IgE in the pathogenesis of urticarial vasculitis remains to be fully elucidated, these findings support the use of omalizumab in selected patients with UV. Larger, prospective controlled studies with longer follow-up are warranted to confirm these results and to further clarify its role in clinical practice.

## ORCID ID

Melek Aslan Kayiran: 0000-0003-4347-3134

Kubra Akdemir Yucel: 0009-0004-4463-1514

Bengu Cobanoglu Simsek: 0000-0003-2639-2017

Mehmet Salih Gurel: 0000-0002-7031-6516

Ethics statement

Ethical approval for this retrospective study was obtained from the Istanbul Medipol University Non-Interventional Research Ethics Committee (Approval date: 01/08/2024; Approval number: E-10840098-202.3.02-4635). The study was conducted in accordance with the principles of the Declaration of Helsinki. As this was a retrospective study, informed consent was waived.

## Financial support

None declared.

## Authors’ contributions

Melek Aslan Kayiran: Study conception and planning; data collection, analysis and interpretation; effective participation in research orientation; preparation and writing of the manuscript; critical literature review; manuscript critical review; approval of the final version of the manuscript.

Kubra Akdemir Yucel: Data collection, analysis and interpretation; preparation and writing of the manuscript; critical literature review; approval of the final version of the manuscript.

Bengu Cobanoglu Simsek: Data collection, analysis and interpretation; intellectual participation in propaedeutic and/or therapeutic management of studied cases; approval of the final version of the manuscript.

Mehmet Salih Gurel: Study conception and planning; manuscript critical review; critical literature review; approval of the final version of the manuscript.

## Research data availability

The entire dataset supporting the results of this study was published in this article.

## Conflict of Interest

None declared.
